# The Metric System

**Published:** 1880-02

**Authors:** 


### THE METRIC SYSTEM.

At the last meeting of The Buffalo Medical Club, a committee, appointed at a previous meeting to investigate and report upon the advisability of the members of the Club adopting Metric weights and measures in the writing of prescriptions, reported favorably. After a thorough discussion, the members of the Club pledged themselves to the exclusive use of the Metric System, for at least one month subsequent to the 1st of February. The decision of the club, as to its permanent adoption, will depend upon the practical experience thus obtained.

The important advantage of a simple relation between the units of weight and the units of measure, and the recognized advantages of a decimal system, which has caused its adoption by all scientific men, and by the profession in Europe, will sooner or later necessitate its general use the world over. We are glad to see the Medical Club—an association of the younger physicians of this city—taking the lead in this matter.

